# The disease burden of migraine patients receiving prophylactic treatments in Korea: a population-based claims database analysis

**DOI:** 10.1186/s12913-022-08191-z

**Published:** 2022-07-12

**Authors:** Seonyoung Park, Sola Han, Hae Sun Suh

**Affiliations:** 1 College of Pharmacy, Pusan National University, Busan, South Korea; 2 Department of Regulatory Science, Kyung Hee University, Seoul, South Korea; 3 College of Pharmacy, Kyung Hee University, Seoul, South Korea; 4 College of Pharmacy, University of Texas at Austin, Austin, TX USA

**Keywords:** Migraine, Disease burden, Prophylactic treatment, Cost, Healthcare resource utilization, Korea

## Abstract

**Background:**

Despite guideline recommendations, the limited benefits and failure of prophylactic treatment in patients with migraine have been reported. This study aimed to estimate the incremental burden (i.e., healthcare resource use and cost) of disease in patients who received at least one prophylactic treatment compared to those who did not.

**Methods:**

This study analyzed the Health Insurance Review and Assessment Service database, which covers the entire population of Korea from December 2014 to November 2019. We included adult patients with migraine (≥18 years) who had ≥1 claim with migraine diagnosis (G43) or received ≥1 prescription of triptan or ergotamine between December 2015 and November 2018. We defined two groups: (1) migraine patients who received at least one prophylactic treatment (prophylaxis group) and (2) migraine patients who never received prophylactic treatments (non-prophylaxis group). We performed propensity score matching to balance the baseline covariates between the two groups. In a matched cohort, we estimated healthcare resource use and costs in terms of outpatient visits, outpatient visits to neurologists, emergency department (ED) visits, and hospitalizations.

**Results:**

After matching, 633,709 and 633,709 patients were identified in the prophylaxis and non-prophylaxis groups, respectively. The healthcare resource utilization was significantly higher in the prophylaxis group than in the non-prophylaxis group in terms of the number of outpatient visits (2.34 vs 1.70), outpatient visits to neurologists (2.23 vs 1.61), ED visits (1.07 vs 1.05), and hospitalizations (1.12 vs 1.09) (all *P* <  0.05). The estimated annual costs per patient were significantly higher in the prophylaxis group than in the non-prophylaxis group for outpatient (102.37 USD vs. 62.46 USD), neurology outpatient (141.80 USD vs. 120.30 USD), and ED visits (550.51 USD vs. 234.14 USD) and hospitalization (817.01 USD vs. 645.97 USD) (all *P* <  0.001).

**Conclusions:**

Migraine patients who received ≥1 prophylactic treatment had a higher burden of disease than migraine patients who received no prophylaxis. This indicates that despite migraine prophylaxis, the migraine-related disease burden remains high, and more efficient migraine prophylaxis strategies are needed.

## Background

Migraine is a chronic neurological disease characterized by periodic pulsatile headaches and accompanying symptoms such as photophobia, phonophobia, nausea, and vomiting [1]. Migraine has a prevalence of 14.4% worldwide, and its prevalence in Korea is estimated to be 25.9% in women and 12.8% in men [2, 3]. According to the 2016 Global Burden of Disease study, migraines are the primary cause of years lived with disability worldwide in both men and women aged 15–49 years, demonstrating that the burden is higher in major productivity groups [4]. Migraine not only reduces the quality of life but also causes a great social and economic burden [5].

Since migraine headache and their accompanying symptoms can place a significant burden on the patients and affect daily functioning and quality of life during and between migraine attacks, appropriate treatment should be necessary. The treatment of migraine includes acute and prophylaxis treatment, especially in patients with frequent severe headaches, both treatments are required [6, 7]. The aim of the acute treatment is to reduce the pain, accompanying symptoms, and disorders associated with migraine attacks. The aim of the prophylaxis treatment is to reduce attack frequency, severity, duration, disability, and overall cost associated with migraine and to improve function and health-related quality of life [8]. Prophylaxis treatment may be offered in any of the following situations: the attacks interfere with daily activities despite acute treatment; high or increasing attack frequency; contraindication to failures, or overuse of acute treatments; adverse effects with acute treatments; and patient preference [8]. Korean and American Headache Society guidelines recommend prophylactic treatments to reduce the burden of migraines and number of attacks [8, 9]. However, prophylactic treatments are still underutilized in patients who appear to be clear candidates [10]. Wang et al. reported that despite 87.5% of migraine patients taking acute medications, only 29.2% of the patients took prophylactic medications, and 68.2% of migraine patients who had not received prophylactic treatment needed prophylactic treatment [11]. Moreover, Delussi et al. reported 30.6% of migraine patients who received prophylactic treatments dropped out because of adverse events which was sedation, paresthesia, tachycardia, arrythmia, irritability, insomnia, weight gain, hypotension, bradycardia. Also, the drugs used for migraine prophylactic treatments (beta-blockers, calcium channel blockers, antidepressants, integrators, antiepileptics, sartans) demonstrated low efficacy [12].

Although assessing the migraine disease burden and effect of prophylactic treatment on patients has garnered increasing attention, studies using real-world data are scarce, and studies observing the burden of migraine among patients receiving prophylactic treatments are lacking.

This study aimed to estimate the incremental burden (i.e., healthcare resource utilization and cost) of disease in those receiving migraine prophylactic treatments compared with those not receiving prophylactic treatments using nationwide population-based claims data.

## Methods

### Data source

This study used the Health Insurance Review and Assessment Service (HIRA) database, which contains national health insurance claims data in Korea from December 1, 2014, to November 30, 2019. The health insurance system in Korea covers approximately 98% of the overall Korean population [13]. The HIRA data that we used included patients’ diagnosis, treatment, procedures, surgical history, cost information on all medical services, and prescription drugs that are reimbursed by the health insurance authority.

### Study population

**Fig. 1:**
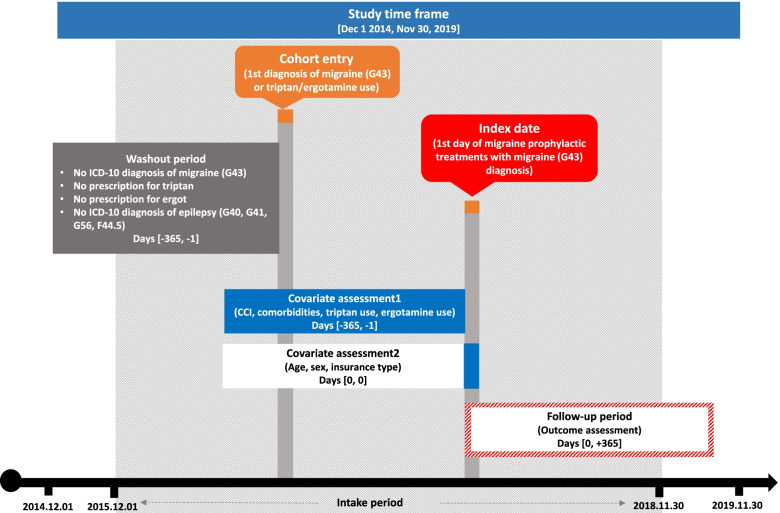
Study scheme

The study scheme is shown in Fig. 1. Adult patients (≥18 years) were identified as migraine patients if they met any of the following inclusion criteria between December 1, 2015, and November 30, 2018: (1) having either primary or secondary diagnosis of migraine (International Classification of Diseases, Tenth Revision [ICD-10] code G43); or (2) having at least one prescription claim for either triptan or ergotamine. The first identified migraine diagnosis or prescription claim for triptan/ergotamine was the cohort entry date. Patients were not included in the study if they met any of the following exclusion criteria: (1) having a diagnosis of migraine (ICD-10 code G43); (2) having at least one prescription claim for either triptan or ergotamine; and (3) having a diagnosis of epilepsy or seizure (ICD-10 codes G40, G41, G56, or F44.5) within 1 year prior to the cohort entry date.

We then defined migraine patients with prophylactic treatments (i.e., prophylaxis group) and patients without prophylactic treatments (i.e., non-prophylaxis group). The prophylaxis group was defined as patients who received at least one prophylactic treatment. The non-prophylaxis group was defined as patients who did not receive any prophylactic treatment. We included the most frequently used prophylactic medications that were selected in Korean clinical practice based on the clinical practice guidelines and the opinions of Korean neurologists [8, 9, 14]. A list of prophylactic medications with anatomical therapeutic chemical codes (ATC codes) is provided in Table 1. Since prophylactic treatments are non-specific drugs for migraine, it is difficult to confirm whether they were prescribed for migraine prophylaxis based on the claims data. Therefore, only the claims that prescribed prophylactic treatments with a migraine diagnosis code (ICD-10 code G43) were included in the analysis according to the opinions of Korean neurologists that if a prophylaxis drug was prescribed with a migraine diagnosis code, it could be judged that the drug was prescribed for migraine prophylaxis.

**Table 1:** Medication codes for migraine prophylactic medications

General name	ATC code*
Divalproex sodium	N03AG01
Valproate	N03AG01
Metoprolol	C07AB02
Propranolol	C07AA05
Atenolol	C07AB03
Nebivolol	C07AB12
Amitriptyline	N06AA09
Topiramate	N03AX11
Venlafaxine	N06AX16
Nadolol	C07AA12
Flunarizine	N07CA03
Cinnarizine	N07CA02
Nortriptyline	N06AA10
Levetiracetam	N03AX14
Zonisamide	N03AX15
Verapamil	C08DA01
Nimodipine	C08CA06
Gabapentin	N03AX12

* *ATC code* Anatomical Therapeutic Chemical code

To identify the prophylaxis group, the index date was defined as the date of claim of the first observed prophylactic treatments with migraine diagnosis (ICD-10 code G43). To identify the non-prophylaxis group, the index date was defined as the same date as the matched prophylaxis patient. Detailed information on the matching process is described in the statistical analyses section. The follow-up period for both groups was 1 year from the index date.

### Migraine-related healthcare resource utilization

In this study, we assessed the frequency of healthcare resource use in migraine patients receiving prophylaxis during a 1-year follow-up period. Healthcare resource utilization included outpatient visits, outpatient visits to neurologists, emergency department (ED) visits, and hospitalizations. Healthcare resource utilization was limited to migraine-related visits. To define migraine-related visits, a migraine diagnosis code (ICD-10 code G43) in either primary or secondary diagnosis was required for each healthcare resource utilization. Neurologist visits were defined as an outpatient visit in which the patients visit a neurologist. ED visits were defined using procedure codes V1100, V1200, V1210, V1220, V1300, V1310, V1320, and V1400 which were the codes for emergency medical management charges from the HIRA database.

### Migraine-related healthcare costs

We evaluated migraine-related healthcare costs during the 1-year follow-up period. Migraine-related healthcare costs included the cost of each type of visit (e.g., outpatient visits, outpatient visits to neurologists, ED visits, and hospitalizations). Migraine-related healthcare costs were derived from claims attributed to outpatient visits, outpatient visits to neurologists, ED visits, and hospitalizations with a migraine diagnosis (ICD-10 code G43). We estimated the mean annual cost per patient (cumulative costs of events/number of patients visiting each healthcare resource). The costs of outpatient visits consisted of medical expenses and medication costs. Medical expenses were defined as total costs, excluding medication costs. All costs are expressed in 2019 US dollars (1 USD = 1156.40 Korean won).

### Statistical analyses

We analyzed the study population from December 1, 2014, to November 30, 2019, and described baseline characteristics such as age, sex, insurance type on the index date, Charlson Comorbidity Index (CCI) score, comorbidities, and co-medications during the year before the index date.

To estimate the incremental burden associated with migraine prophylaxis, propensity score matching was conducted between the prophylaxis and non-prophylaxis groups (controls). Propensity score matching was used to minimize potential confounding effects arising from differences in baseline covariates. The propensity score was calculated using logistic regression by including the following variables: sex, age, insurance type, CCI index, comorbidities, and co-medications). Comorbidities in the propensity score model were based on previous studies [15, 16] and included the following: depression (ICD-10 codes F32 and F33), anxiety (ICD-10 code F41), reactions to severe stress and adjustment disorders (ICD-10 code F43), sleep disorder (ICD-10 code F51), sinusitis (ICD-10 code J01), upper respiratory infections (ICD-10 code J06), bronchitis (ICD-10 code J20), dorsopathies (ICD-10 code M53), dorsalgia (ICD-10 code M54), and dyspepsia (ICD-10 code K30). The co-medications in the propensity score model included triptans and ergotamine to balance the frequency of migraine attacks within 1 year prior to the index date. We used one-to-one greedy matching, in which cases were matched sequentially with controls with the closest propensity score. The balance of baseline covariates between the two groups was assessed using standardized differences in the matched samples, and standardized differences of less than 10% were considered acceptable.

Study variables are summarized as counts with percentages for categorical variables and as mean and standard deviations for continuous variables. Chi-squared tests were used for categorical variables, and Student’s t-test was used for continuous variables. All statistical analyses were performed using SAS Enterprise Guide 7.1 (SAS Institute Inc., Cary, North Carolina, USA). We considered a result to be statistically significant when the *p*-value was less than 0.05.

## Results

### Sample selection

**Fig. 2:**
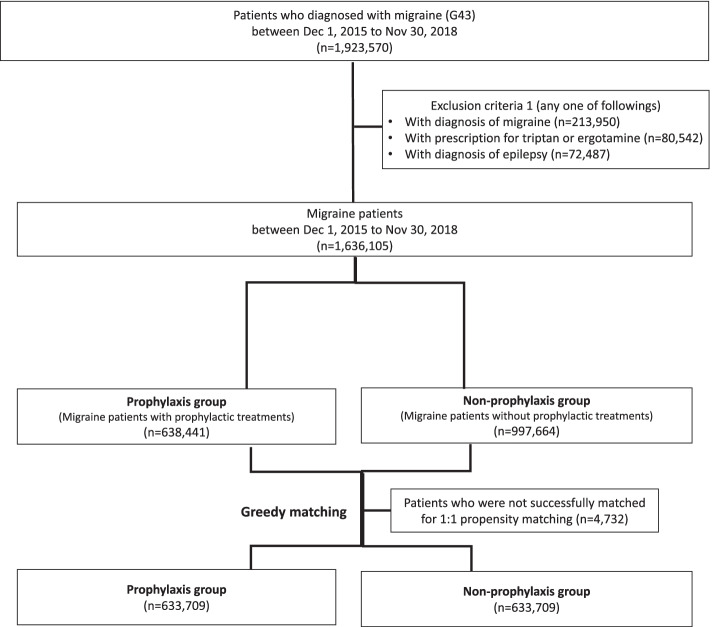
Flow chart of study sample selection

Between December 1, 2015, and November 30, 2018, a total of 1,636,105 patients with a diagnosis of migraine or prescription for triptan or ergotamine were defined as migraine patients. We identified 638,441 migraine patients who received prophylactic treatments and 997,664 migraine patients who did not receive prophylactic treatment. After matching, 633,709 patients in the prophylaxis group and 633,709 patients in the non-prophylaxis group were selected (Fig. 2).

### Baseline characteristics

The baseline characteristics of the unmatched cohorts are shown in Table 2. Before matching, the prophylaxis group had a higher age, CCI index, and higher prevalence of medical comorbidities than those of the non-prophylaxis group. The prophylaxis group had higher triptan and ergotamine use than the non-prophylaxis group. The absolute standardized differences were greater than 0.1 for single episode major depressive disorder, anxiety, and dorsalgia before matching (Table 2). The baseline characteristics of the matched cohorts are shown in Table 3. The mean age was 49.04 years, and 69.74% of patients were female in the matched cohort. Triptan and ergotamine use were similar in the matched cohort. After matching, all absolute standardized differences were less than 0.1.

**Table 2:** Baseline characteristics of prophylaxis and non-prophylaxis groups in unmatched cohort

Unmatched cohort				
	Prophylaxis group (*N* = 638,441)	Non-prophylaxis group (*N* = 997,664)	*P*-value*	Absolute standardized difference
**Age in years, mean (SD)**	49.00 (16.90)	48.25 (17.47)	< 0.001	0.04
**Sex, No. (%)**				
Male	192,587 (30.17)	333,657 (33.44)	< 0.001	−0.07
Female	445,854 (69.83)	664,007 (66.56)		
**Insurance type, No (%)**				
National Health Insurance program	611,578 (95.79)	957,794 (96.00)	< 0.001	−0.01
Medical aid	26,654 (4.17)	39,261 (3.94)		
**Charlson Comorbidity Index, mean (SD)**	1.47 (1.72)	1.30 (1.68)	< 0.001	0.1
**Comorbidities, No (%)**				
Major depressive disorder, single episode	77,058 (12.07)	85,091 (8.53)	< 0.001	0.12
Major depressive disorder, recurrent	6268 (0.98)	7252 (0.73)	< 0.001	0.03
Other anxiety disorders	135,537 (21.23)	153,141 (15.35)	< 0.001	0.15
Reaction to severe stress and adjustment disorders	11,232 (1.76)	13,364 (1.34)	< 0.001	0.03
Sleep disorders not due to a substance or known physiological condition	41,749 (6.54)	52,976 (5.31)	< 0.001	0.05
Acute sinusitis	111,703 (17.50)	157,676 (15.80)	< 0.001	0.05
Acute upper respiratory infections of multiple and unspecified sites	180,798 (28.32)	262,532 (26.31)	< 0.001	0.05
Acute bronchitis	365,723 (57.28)	531,149 (53.24)	< 0.001	0.08
Other and unspecified dorsopathies, not elsewhere classified	31,926 (5.00)	41,017 (4.11)	< 0.001	0.04
Dorsalgia	279,153 (43.72)	383,924 (38.48)	< 0.001	0.11
Dyspepsia	268,219 (42.01)	378,967 (37.99)	< 0.001	0.08
**Number of uses of triptan, mean (SD)**	0.37 (5.08)	0.47 (5.43)	< 0.001	−0.02
**Number of uses of ergotamine, mean (SD)**	0.52 (8.97)	0.96 (11.47)	< 0.001	− 0.04

*SD* standard deviation.

* Chi-squared test and Student’s t-test were used to compare prophylaxis and non-prophylaxis groups

**Table 3:** Baseline characteristics of prophylaxis and non-prophylaxis groups in matched cohort

	Matched cohort			
	Prophylaxis group (*N* = 633,709)	Non-prophylaxis group (*N* = 633,709)	*P*-value*	Absolute standardized difference
**Age in years, mean (SD)**	49.04 (16.91)	49.04 (16.96)	0.97	− 0.00006
**Sex, No. (%)**				
Male	191,749 (30.26)	191,749 (30.26)	1.00	0.000
Female	441,960 (69.74)	441,960 (69.74)		
**Insurance type, No (%)**				
National Health Insurance program	607,088 (95.80)	607,692 (95.89)	0.009	−0.005
Medical aid	26,412 (4.17)	25,842 (4.08)		
**Charlson Comorbidity Index, mean (SD)**	1.47 (1.72)	1.46 (1.72)	0.08	0.003
**Comorbidities, No (%)**				
Major depressive disorder, single episode	75,245 (11.87)	74,501 (11.76)	0.04	0.003
Major depressive disorder, recurrent	6143 (0.97)	5608 (0.88)	< 0.001	0.009
Other anxiety disorders	132,771 (20.95)	134,017 (21.15)	0.007	−0.005
Reaction to severe stress and adjustment disorders	10,966 (1.73)	10,766 (1.70)	0.17	0.002
Sleep disorders not due to a substance or known physiological condition	41,126 (6.49)	41,115 (6.49)	0.97	0.0001
Acute sinusitis	110,284 (17.40)	109,915 (17.34)	0.39	0.002
Acute upper respiratory infections of multiple and unspecified sites	178,928 (28.24)	178,950 (28.24)	0.97	−0.0001
Acute bronchitis	362,187 (57.15)	363,553 (57.37)	0.01	−0.004
Other and unspecified dorsopathies, not elsewhere classified	31,349 (4.95)	30,542 (4.82)	< 0.001	0.006
Dorsalgia	275,789 (43.52)	275,928 (43.54)	0.8	−0.0004
Dyspepsia	265,291 (41.86)	265,901 (41.96)	0.27	−0.002
**Number of uses of triptan, mean (SD)**	0.37 (5.09)	0.35 (4.50)	0.02	0.004
**Number of uses of ergotamine, mean (SD)**	0.52 (8.99)	0.63 (9.22)	< 0.001	−0.012

*SD* standard deviation.

* Chi-squared test and Student’s t-test were used to compare prophylaxis and non-prophylaxis groups

### Migraine-related healthcare resource utilization

Migraine-related visits were assessed during the 1-year follow-up period (Table 4). Migraine-related healthcare resource utilization was significantly higher in the prophylaxis group than in the non-prophylaxis group. The mean number of migraine-related outpatient visits per patient was 2.34 (SD = 3.00) in the prophylaxis group and 1.70 (SD = 2.13) in the non-prophylaxis group (*P* <  0.001). The mean number of migraine-related outpatient visits to neurologists per patient was 2.23 (SD = 2.26) in the prophylaxis group and 1.61 (SD = 1.20) in the non-prophylaxis group (*P* <  0.001). Although the difference was small, the mean numbers of migraine-related hospitalizations (1.12 vs. 1.09; *P* <  0.001) and ED visits (1.07 vs. 1.05; *P* <  0.001) were also significantly higher in the prophylaxis group than in the non-prophylaxis group.

**Table 4:** Migraine-related healthcare resource utilization in the prophylaxis and non-prophylaxis groups

	Prophylaxis group (*N* = 633,709)	Non-prophylaxis group (*N* = 633,709)	*P*-value*
Outpatient visits, mean (SD)	2.34 (3.00)	1.70 (2.13)	< 0.001
Neurologist visits, mean (SD)	2.23 (2.26)	1.61 (1.20)	< 0.001
ED visits, mean (SD)	1.07 (0.37)	1.05 (0.39)	0.0025
Hospitalizations, mean (SD)	1.12 (1.39)	1.09 (0.55)	0.01

*SD* Standard Deviation.

* Student’s t-test were used to compare prophylaxis and non-prophylaxis groups

### Migraine-related healthcare costs

Table 5 shows mean migraine-related healthcare costs per patient for outpatient visits, outpatient visits to neurologists, ED visits, and hospitalizations during the 1-year follow-up period. In the prophylaxis group, mean outpatient costs per patient were 102.37 USD, with medical expenses accounting for 68.73 USD and medication costs accounting for 33.65 USD. In the non-prophylaxis group, mean outpatient costs per patient were 62.46 USD, with medical expenses accounting for 43.81 USD and medication costs accounting for 18.65 USD. The mean costs for outpatient visits to neurologists per patient were significantly higher in the prophylaxis group than in the non-prophylaxis group (141.80 USD vs. 120.30 USD; *P* <  0.001). The mean costs of ED visits (550.51 USD vs. 234.14 USD) and hospitalizations (817.01 USD vs. 645.97 USD) were also significantly higher for the prophylaxis group than for the non-prophylaxis group. Throughout the 1-year follow-up period, all migraine-related healthcare costs per patient in the prophylaxis group were greater than those in the non-prophylaxis group.

**Table 5:** Migraine-related healthcare costs of the prophylaxis and non-prophylaxis groups

(Unit: USD)							
	Prophylaxis group (*N* = 633,709)			Non-prophylaxis group (*N* = 633,709)			*P*-value*
	Mean (%)	Median	SD	Mean (%)	Median	SD	
**Outpatient visits (per patient)**							
Total visits							
Total outpatient cost	102.37	38.74	239.23	62.46	25.07	137.62	< 0.001
Medical expense	68.73(67.13)	25.58	142.51	43.81 (70.14)	16.53	104.65	< 0.001
Medication cost	33.65 (32.87)	6.22	171.96	18.65 (29.86)	3.88	72.84	< 0.001
Neurologist visits							
Total cost	141.80	64.34	339.38	120.30	49.78	215.46	< 0.001
Medical expense	85.57 (60.35)	33.75	137.67	82.37 (68.47)	24.71	175.90	0.01
Medication cost	56.23 (39.65)	10.70	294.32	37.93 (31.53)	5.61	113.25	< 0.001
**ED visits (per patient)**							
Total ED cost	550.51	269.35	1055.11	234.14	175.54	435.65	< 0.001
Medical expense	549.81 (99.87)	268.78	1055.08	233.71 (99.81)	175.35	435.58	< 0.001
Medication cost	0.70 (0.13)	0	4.83	0.44 (0.19)	0	2.89	< 0.001
**Hospitalizations (per patient)**							
Hospitalization cost	817.01	482.56	1342.32	645.97	347.19	1212.88	< 0.001

*SD* standard deviation.

* Chi-squared test and Student’s t-test were used to compare prophylaxis and non-prophylaxis groups

## Discussion

This is the first study to estimate the incremental disease burden incurred by the migraine prophylaxis group compared with that of the non-prophylaxis group in Korea, based on national health insurance claims data. To compare the two groups, propensity score matching was conducted to balance the baseline characteristics and minimize selection bias.

In this retrospective study using the Korea national health insurance claims data, 1,636,105 migraine patients were identified, of whom 638,441 patients (39%) received at least one migraine prophylactic treatment. In two previous studies, 33.8 and 38.8% of migraine patients were estimated to require prophylactic treatment, which is consistent with our findings [10, 17].

In this study, the mean annual number of migraine-related outpatient visits per patient was 2.34 and 1.70, respectively. Similar trends were observed in the number of outpatient visits among migraine patients in Finland. The number of outpatient visits was 2.4 per patient-year in migraine patients receiving prophylactic treatments and 1.3 per patient-year in migraine patients only receiving acute treatment [15]. A cross-sectional analysis of survey data from France, Germany, Italy, Spain, and the United Kingdom estimated healthcare resource utilization from visits to the healthcare system 6 months before survey participation [18]. In this study, they reported the mean number of outpatient visits to neurologists was 0.19, and the mean number of ED visits was 0.46 in migraine patients. The estimated numbers of visits were lower than those reported in our study (2.23 outpatient visits to neurologists and 1.07 ED visits per patient-year). This may reflect differences in access to healthcare. Most European countries offer gatekeeping systems in which patients first see a general practitioner before a specialized physician; however, in Korea, patients can see a specialized physician directly without having to see a general practitioner [19, 20].

Yu et al. reported that the mean annual outpatient cost per patient was 46.5 USD among migraine patients in China [3]. In the current study, the mean annual outpatient costs were 102.37 USD in the prophylaxis group and 62.46 USD in the non-prophylaxis group. Migraine patients receiving prophylactic treatment were not included in the Chinese study. Thus, it was difficult to make an accurate comparison; however, in the non-prophylaxis group, the mean annual outpatient costs were similar to those in the study from China.

The current study did not observe the effect of migraine prophylactic treatments but instead evaluated the present status of disease burden in patients receiving migraine prophylactic treatments. These study findings indicate that migraine-related healthcare resource utilization and healthcare costs were significantly higher in migraine patients who received at least one migraine prophylactic treatment than in those who never received migraine prophylactic treatment. Although international guidelines recommend prophylactic treatment to reduce the burden of migraine and number of migraine attacks, prophylactic treatments for migraine remained underutilized in patients who appear to be clear candidates [10]. Moreover, most migraine patients who receive prophylactic treatments dropped out because of adverse events and the low efficacy of drugs [12]. Therefore, these findings suggest that despite the use of migraine prophylactic treatments, there are still unmet medical needs in the migraine patients who received prophylactic treatment. These results are consistent with those of previous studies investigating the effect of migraine prophylactic treatment and estimating the burden of unmet medical needs in migraine patients [12, 21]. As a result, these findings reveal that more effective strategies and treatments to prevent migraine attacks are needed to reduce the burden of migraine patients receiving prophylactic treatment. In addition, we tried to evaluate the disease burden in patients who received at least one prophylactic medication in Korea overall. Further studies examining the disease burden according to the number of classes of prophylactic treatment may be required to provide more information on the disease burden and unmet needs of current prophylactic treatment.

The current study has several limitations. First, we used propensity score matching to minimize potential confounding effects on incremental disease burden. Although we accounted for measured confounders in the matching process, unmeasured confounders, such as monthly migraine days and clinical data representing the severity of migraine, which were not included in the claims data, were not considered and may have affected the analysis. However, we considered the use of triptan and ergotamine in the matching process to balance the severity of migraine between the two groups. Second, in this study, we evaluated the present status of disease burden in patients receiving migraine prophylactic treatments in Korea but did not observe the treatment patterns and dropouts. Treatment discontinuation that may be due to adverse effects or lack of efficacy may have occurred during the follow-up period and the compliance of the treatment was not considered. A previous study reported that prophylactic treatments were associated with a high rate of discontinuation due to adverse effects or lack of efficacy [15, 22]. Thus, further studies focusing on the dropouts and treatment patterns of prophylactic treatments would be needed to overcome the limitations of this research with more abundant data. Third, since the migraine prophylactic medications used in this study are migraine non-specific medications, some prophylactic medications could have been prescribed for other indications [23]. Thus, it may cause overestimation of certain treatments and costs. To minimize this probability, we selected the most frequently used prophylactic treatments in Korean clinical practice based on clinical expert opinions of Korean neurologists. In addition, we only included claims with a migraine diagnosis code (ICD-10 code G43) in the analysis. Fourth, since many migraine patients in Korea are underdiagnosed and undertreated, a significant proportion of migraine patients in Korea might not be included in current study. Korean migraine patients have unmet needs in terms of diagnosis [6]. Kim et al. reported that because the diversity of migraine symptoms makes some physicians feel unsure of the diagnosis of migraine and many patients also take painkillers during the early phase of migraine attacks so that their headaches frequently do not fit the diagnostic criteria of migraine [6]. For this reason, relatively few patients are diagnosed with migraine by physicians. As a result, many underdiagnosed and undertreated migraine patients might not be included in this study because we used claims data. Additionally, because our study focused on patients newly diagnosed with migraine, patients already diagnosed with migraine were not included in this study.

Despite these limitations, this study has several strengths. First, this study showed that a sufficient reduction in the burden of migraine was not observed in patients receiving prophylaxis, although the guidelines recommend preventive therapies to reduce the burden of migraine. It is necessary to examine whether prophylactic treatments are used properly, and more effective treatment strategies are needed. Second, this result is meaningful because evidence regarding disease burden in patients with migraine who received prophylactic treatments is very scarce [15]. In addition, the results of this study are representative and reliable because we used a nationwide claims database that represents approximately 98% of the overall Korean population [13].

## Conclusions

This study assessed the incremental disease burden related to migraine prophylaxis on healthcare resource utilization and healthcare costs among migraine patients who received prophylaxis compared with those who did not in Korea using representative population-based data. This study is significant because it showed that a sufficient reduction in the burden of migraine was not observed in patients with prophylaxis, although guidelines recommended preventive therapies to reduce the burden of migraine. The findings of this study indicate that there are still unmet needs in migraine patients with prophylactic treatments and highlight the need for appropriate prevention strategies for migraine to reduce the burden of disease.

## Data Availability

The study data were extracted and analyzed from the Korea Health Insurance Review & Assessment Service (HIRA) claims database, and data may be obtained from the HIRA with appropriate authorization approval.

## Abbreviations

HIRA: Health Insurance Review and Assessment Service
ED: Emergency Department
ICD-10: International Classification of Diseases, Tenth Revision
ATC: Anatomical Therapeutic Chemical
CCI: Charlson Comorbidity Index
SD: Standard Deviation
